# *Plasmodium falciparum* Thioredoxin Reductase (*Pf*TrxR) and Its Role as a Target for New Antimalarial Discovery

**DOI:** 10.3390/molecules200611459

**Published:** 2015-06-22

**Authors:** Sara E. McCarty, Amanda Schellenberger, Douglas C. Goodwin, Ngolui Rene Fuanta, Babu L. Tekwani, Angela I. Calderón

**Affiliations:** 1 College of Sciences and Mathematics, Auburn University, Auburn, AL 36849, USA; E-Mail: sem0026@auburn.edu; 2 Department of Drug Discovery and Development, Harrison School of Pharmacy, Auburn University, Auburn, AL 36849, USA; E-Mail: ans0038@auburn.edu; 3 College of Agriculture, Auburn University, Auburn, AL 36849, USA; 4 Department of Chemistry and Biochemistry, Auburn University, Auburn, AL 36849, USA; E-Mails: goodwdc@auburn.edu (D.C.G.); rfn0001@tigermail.auburn.edu (N.R.F.); 5 National Center for Natural Products Research and Department of BioMolecular Sciences, School of Pharmacy, University of Mississippi, Oxford, MS 38677, USA; E-Mail: btekwani@olemiss.edu

**Keywords:** thioredoxin reductase, *Plasmodium falciparum*, malaria, animal models

## Abstract

The growing resistance to current antimalarial drugs is a major concern for global public health. The pressing need for new antimalarials has led to an increase in research focused on the *Plasmodium* parasites that cause human malaria. Thioredoxin reductase (TrxR), an enzyme needed to maintain redox equilibrium in *Plasmodium* species, is a promising target for new antimalarials. This review paper provides an overview of the structure and function of TrxR, discusses similarities and differences between the thioredoxin reductases (TrxRs) of different *Plasmodium* species and the human forms of the enzyme, gives an overview of modeling *Plasmodium* infections in animals, and suggests the role of Trx functions in antimalarial drug resistance. TrxR of *Plasmodium falciparum* is a central focus of this paper since it is the only *Plasmodium* TrxR that has been crystallized and *P. falciparum* is the species that causes most malaria cases. It is anticipated that the information summarized here will give insight and stimulate new directions in which research might be most beneficial.

## 1. Introduction

Malaria, a deadly disease characterized by high fevers and chills, is caused by *Plasmodium* spp*.* protozoan parasites that are transmitted to humans via a bite from an infected *Anopheles* mosquito [1]. An estimated 198 million cases of malaria occurred worldwide in 2013 with approximately 584,000 deaths. Four parasite species are known to cause malaria in humans, *P. falciparum*, *P. vivax*, *P. malariae*, and *P. ovale*, while a fifth species that mainly infects monkeys, *P. knowlesi*, has recently been found in human cases of malaria as well [2]. Most malaria cases and deaths are caused by *P. falciparum*, and *P. falciparum* and *P. vivax* pose the greatest challenges to public health [3]. *P. ovale* and *P. vivax* in humans and *P. cynomolgi* in monkeys can cause recurrent malaria due to their ability to persist in the liver of hosts in a dormant hypnozoite form [4,5]. *Plasmodium* species have three stages in their life cycle: the pre-erythrocytic cycle, which occurs in the vector and then the human liver, the erythrocytic cycle, which occurs in human red blood cells, and the sporogonic cycle, which occurs mainly in the insect vector [6]. The erythrocytic stage is primarily responsible for pathogenesis of malaria and is most harmful to the host [7].

*P. falciparum* relies on two antioxidant systems to relieve oxidative stress and maintain redox equilibrium within the parasite [8]. These are the glutathione and thioredoxin systems. The thioredoxin system consists of thioredoxin reductase (TrxR), its substrate, thioredoxin (Trx), and NADPH. TrxR contains a dithiol/disulfide active site and serves as a cellular protein disulfide reductase, while Trx is an electron donor for a multitude of enzymes, including ribonucleotide reductases, thioredoxin peroxidases and methionine sulfoxide reductases [9].

Recently, reviews have been written covering *Plasmodium*’s antioxidant systems describing in detail how TrxR works as known at that time [10], comparing antioxidant systems of parasites, including *P. falciparum*, in order to further understanding of parasite biology, parasite-host interactions, and drug resistance [11], reviewing the current knowledge on thioredoxin superfamily and its role in *Plasmodium* [12], and using the thioredoxin system or TrxR as a plausible drug target [13,14,15,16,17,18,19,20,21].

This review presents an overview of *Pf*TrxR structure and functions, especially comparison with TrxR identified in different rodent and primate *Plasmodium* spp., and potential role of TrxR in antimalarial drug resistance. Additionally, we have identified the areas and potential gaps in the knowledge related to *Plasmodium* TrxR. This would stimulate further research on potential of TrxR as target for antimalarials against *Plasmodium* species that are infectious to humans vis-à-vis design and development of more specific inhibitors of *Pf*TrxR as potential new antimalarial drug leads.

## 2. Key Aspects of *Pf*TrxR Active Sites for Enzyme Inhibition

**Figure 1:**
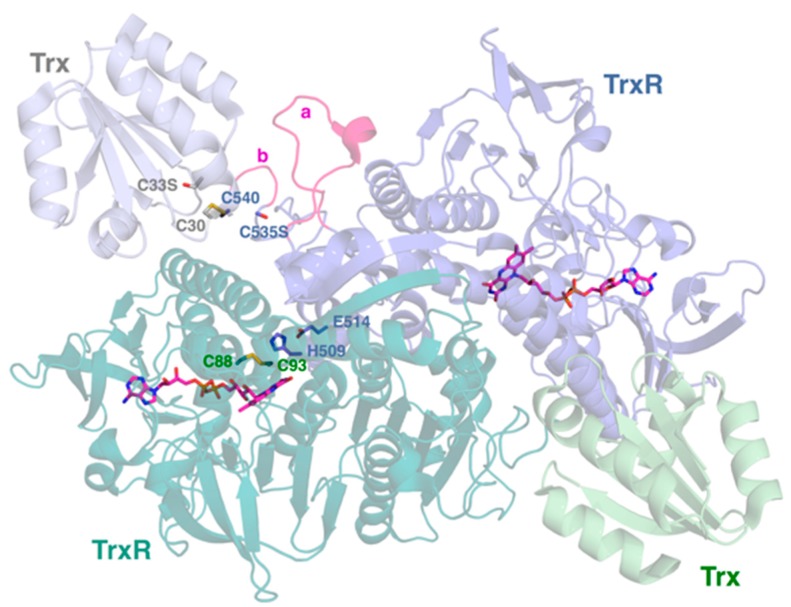
The dimeric structure of *P. falciparum* thioredoxin reductase (TrxR). The two subunits of TrxR are shown with their bound FAD cofactors in magenta. The redox active disulfide (C88–C93) of the N-terminal redox center is shown for the TrxR monomer on the lower left. H509 and E514 that modulate the reactivity of this N-terminal redox center are supplied by the TrxR monomer on the upper right. Each monomer is bound to substrate Trx via intermolecular disulfide (TrxR C540 to Trx C30). The positions of C535 (TrxR) and C33 (Trx) are indicated by serine residues. These substitutions were made in order to trap the intermolecular disulfide between TrxR and Trx [23]. The 535 and 540 residues shown are supplied by the TrxR monomer on the upper right. The *Plasmodium*-unique insertions H438–S452 and G536–K539 are highlighted by *a* and *b*, respectively. Coordinates are drawn from PDB accession 4J56 [23]. The figure was generated using PyMOL 1.6.0.0.

The structure of *Pf*TrxR complexed with its substrate, thioredoxin (Figure 1), has been solved only recently [22,23]. Although *Pf*TrxR shows distinct characteristics in comparison to TrxR identified in other Apicomplexan parasites and the human counterparts (e.g., the extended insertion loop addressed below), there isa striking similarity between the TrxR from different *Plasmodium* species. As with all known TrxR enzymes, *Pf*TrxR is a homodimeric protein where each subunit divides into three major domains. The FAD-binding domain roughly includes residues 38 to 197 and 321 to 390. The residues between the two sequence components of the FAD-binding domain (*i.e.*, 198–320) have been assigned as the NADPH-binding domain, and the C-terminal segment of the subunit from about S391 to the C-terminus (G541) is referred to as the interface domain. The homodimeric structure of *Pf*TrxR contains two fully functional pairs of redox centers. The N-terminal redox center is referred to as such because the bulk of residues for FAD and NADPH binding as well as electron transfer associated with this center are provided by the N-terminal domains (38–320). However, it is important to point out that two residues provided by the C-terminal interface domain of the second subunit (H509 and E514) contribute to the function of this center. The location of the N-terminal dual-cysteine redox center is immediately adjacent to the isoalloxazine ring system of the FAD prosthetic group. The so-called C-terminal redox center is accounted for almost entirely by the C-terminal nine amino acids (G533–G541), most notably cysteines 535 and 540. A notable insertion loop also interacts with both the substrate and the enzyme. The flexible conformation of the C-terminal arm and the extended insertion loop set *Pf*TrxR apart from human TrxR and contribute to differences in substrate binding and reduction [22]. Formation of the enzyme-substrate (*Pf*TrxR-Trx) complex results in rearrangement of the C-terminal active site and the insertion loop, which is a necessary conformational change for the enzyme’s catalytic cycle [23]. This feature of *Pf*TrxR is similar to that of human TrxR.

### 2.1. N-Terminal Redox Center

Four amino acids in the N-terminal domain are essential to TrxR activity: Cys 88, Cys 93, His 509, and Glu 514. Cys 88 and Cys 93 are buried in the protein and are the endpoints of a strictly conserved CVNVGC sequence that forms a single five-amino-acid helical turn [24,25]. This helical turn dramatically distorts what is otherwise a typical alpha helix, and is a typical feature of disulfide reductases. In the enzyme’s oxidized state, Cys 88 and Cys 93 are joined to one another through a disulfide bond. The disulfide resides directly above N1 of the flavin isoalloxazine ring system. [23,25]. Replacement of either Cys 88 or Cys 93 with either Ser or Ala results in the complete loss of TrxR activity in reducing DTNB. Cys 93 is implied to be the thiolate flavin charge transfer thiol for the N-terminal redox center after testing conditions stated previously [25]. His 509 and Glu 514 are known to be essential to the active site and are contributed by a strongly conserved region of the interface domain of the second subunit. The ε2 nitrogen of the histidine imidazole is in closest proximity to the sulfur of Cys 88. Simultaneously, the δ1 nitrogen of the His 509 imidazole is in H-bonded contact with the Glu 514 carboxylate [23]. Thus, it is proposed that these two residues form a catalytic dyad where the general acid/base properties of His 509 are modulated by Glu 514 [25]. In support of this idea, substitution of His 509 with either Gln or Ala decreases the *Pf*TrxR *k*_cat_ for NADPH-dependent DTNB reduction by two orders of magnitude with relatively small impact on the apparent *K*_M_ values for either substrate [25]. Similarly, substitution of Glu 514 with Ala produces an order of magnitude decrease in activity [25]. In any case, residual albeit minimal activity in H509A variants suggest that another residue may be able to substitute for His 509 to a limited extent in catalysis [25,26].

### 2.2. C-Terminal Redox Center

The C-terminal redox center is found on the highly conformationally flexible C-terminus of the interface domain. In contrast to mammalian TrxR C-terminal redox center, which is composed of immediately adjacent cysteine-selenocysteine residues as a redox pair (G***CU***G-COO^−^), *Pf*TrxR employs a two-cysteine redox pair (Cys 535 and Cys 540) where the Cys residues are separated by an intervening four-amino-acid linker (G***C***GGGK***C***G-COO^−^) [22]. Several amino acids and a disulfide ring contribute to the active site of the C-terminal redox center. Cys 535 is hydrogen bonded to neighboring amino acids on the flexible arm of the terminal domain. Cys 535 has several important functions including electron transfer, reduction of thioredoxin and serving as the resolving cysteine of the intermolecular disulfide. Cys 540 is also involved in electron transfer and reduction of the substrate, but it has also been suggested to be the most physiologically relevant nucleophilic cysteine of the C-terminus. This is due to its flexibility, since it is located on the flexible arm of the C terminus, and has been determined to be the ideal nucleophile to approach the substrate [23]. The C-terminal amino acid (Gly 541) affects enzymatic activity by forming a salt bridge with Lys58 with its backbone carboxylate as well as a hydrogen bond between its amide nitrogen and the carbonyl oxygen of Gly 534 [24]. The hydrogen bond is more important in stabilizing the β-turn-β motif and serves as an anchor for the reduction of the C-terminal domain disulfide. The C-terminal redox center has a twenty-member disulfide ring when C-terminal Cys residues 535 and 540 are oxidized, similar to the N-terminal extension, which has an impact on activity. Deletion of Lys 539 from between the C-terminus cysteines in the ring, leaving it with only seventeen atoms, has a significant reduction on the enzyme activity. Therefore, C-terminal domain chain length is important to the ring’s functionality [24].

### 2.3. Enzyme Catalytic Cycle

TrxR-catalyzed reduction of Trx by NADPH is proposed to require a priming stage where NADPH binding is followed by formation a FADH^−^ to NADP^+^ charge transfer complex. Along with NADP^+^ release, FADH^−^ is reoxidized at the expense of Cys 93, forming Cys-S^−^ to FAD charge transfer complex and a thiol for subsequent disulfide exchange at Cys 88. This form of TrxR is proposed to be the start and end of the enzyme’s catalytic cycle. Through intersubunit dithiol-disulfide exchange with the disulfide C-terminal redox center, the N-terminal redox center is returned to its fully oxidized state (*i.e.*, FAD, Cys 88–Cys 93 disulfide) and the C-terminal redox center is reduced from its disulfide to dithiol state. In a sequence essentially identical to the priming steps, NADPH binding produces the NADP^+^ to FADH^−^ charge transfer complex which again transitions to a Cys 93 thiolate to FAD charge transfer complex and a Cys 88 thiol. With both the N- and C-terminal redox centers fully reduced, Trx reacts with the C-terminal redox center through dithiol: disulfide exchange to convert the Trx disulfide substrate to the corresponding reduced dithiol Trx product. In so doing, the C-terminal redox center returns to its oxidized disulfide state, and the next reaction cycle can begin [27].

### 2.4. Interactive Cavity and Interface

Within *Pf*TrxR there are a narrow active site cavity and a monomer-monomer interface. In the cavity, Tyr 101 and His 104 alter the stereochemical properties of the cavity in *Pf*TrxR compared to the Gln and Leu in cavity in human thioredoxin reductase (hTrxR). The narrowness is suggested to be due to a H-bond with Asp 112 on the α3 helix from the other monomer. Due to the narrowness of the cavity, it is difficult for larger molecules to access the *Pf*TrxR active site [22,23]. Indeed, it has been suggested that the cavity can host smaller, more amphipathic molecules [22]. The *Pf*TrxR interface is a bent α3 helix because of the unique presence of Met 105 and Phe 109. It contributes to dimer stability and to substrate binding of thioredoxin due to the bulky Met 105 and Phe 109 inducing a bend that goes on to stimulate a conformational change downstream on the same interface. *Pf*TrxR assumes an anti-parallel conformation that allows H-bonding particularly between Asp 121 and Asn 122 [22,23].

### 2.5. Insertion Loop

*Pf*TrxR has an insertion loop of amino acids, His 438-Ser 456, in a cleft within the protein. This loop consists of nineteen residues in *Pf*TrxR*,* but only five residues in hTrxR. Different parts of the loop interact with different substrates or intermolecular residues. Some interact only with residues of *Pf*TrxR, others with the C-terminus in particular, and three interact with reductase and substrate interface residues. Deletion of residues 438–452 of the loop produced a no change in the *K*_M_ for the pseudosubstrate DTNB but a seven fold increase in *K*_M_ for the true substrate, Trx. Interestingly, the *k*_cat_ value with respect to both substrates were only moderately diminished. These data suggest that the insertion loop is important for interaction and complex formation between *Pf*TrxR and its *Pf*Trx substrate. His 438 may be particularly important in reduction of the disulfide bond. The H438A *Pf*TrxR variant can account almost entirely for the decrease in *k*_cat_ observed for the loop deletion variant, but itself shows no impact on the *K*_M_ with respect to *Pf*Trx [23].

*Pf*TrxR has a higher *K*_M_ and lower *k*_cat_ for both *Pf*Trx and DTNB than is observed for hTrxR [23,27,28]. Replacing Cys with Sec in the second position of the redox center, which makes *Pf*TrxR more like hTrxR, shows activities with *E. coli* Trx lower than that of the wild-type *Pf*TrxR. This result may be due to incomplete ligation of the peptide containing Sec during incorporation into the enzyme using a semisynthetic technique. Why *P. falciparum* did not evolve to use Sec is still unknown [24].

## 3. TrxR: Human and *Plasmodium* Species

### 3.1. TrxR Isoenzymes

Mammalian TrxR can be found as three isoenzymes: TrxR1, TrxR2, and TGR. hTrxR1 is the cytosolic form of the enzyme while hTrxR2 is the miotochondrial form. Both hTrxR1 and hTrxR2 have the CVNVGC active site and the FAD-binding domain and NADPH-binding domain common to *Plasmodium* TrxR forms. However, hTrxR2 has a 33-amino acid extension at the N-terminus that is thought to be a mitochondrial translocation signal. A third form, TGR, sometimes referred to as hTrxR3, exists mainly in developing sperm. It is more similar to hTrxR1 than to hTrxR2 and, unlike other TrxRs, has a N-terminal glutaredoxin domain, hence the name “thioredoxin glutathione reductase,” or TGR [18]. Like hTrxR, *Pf*TrxR has an isoform found in the cytosol (*Pf*TrxR1) and one found in the mitochondria (*Pf*TrxR2) [29].

### 3.2. Identities in the Sequences of Human and Plasmodium Species TrxRs

**Figure 2:**
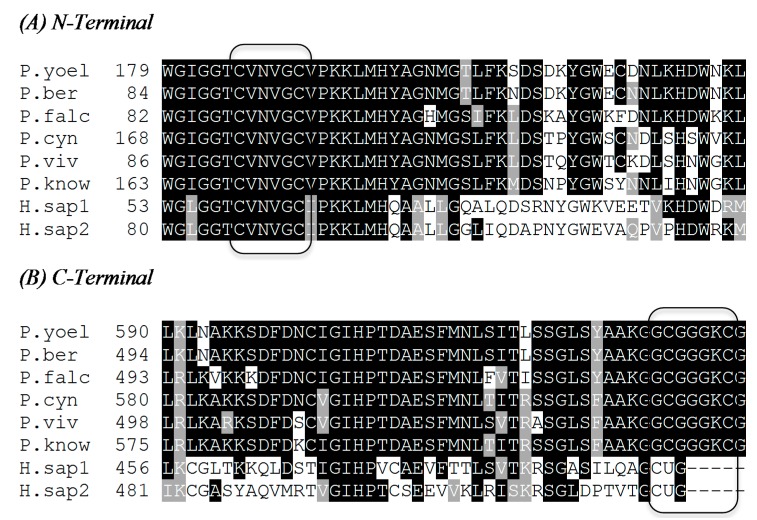
Active site comparison between human and *Plasmodium* species for (**A**) the N-Terminal and (**B**) the C-Terminal. Black shaded amino acids represent identity with the corresponding black shaded amino acids of other species’ TrxR while grey represents amino acids with similar properties and no shading represent no identity. The figure was generated with ClustalW2 MSA (Multiple Sequence Alignment) program.

The predicted amino acid sequences of TrxR from different *Plasmodium spp* were obtained from GenBank*.* Pairwise and multiple sequence alignments were performed with ClustalW and the shaded box plots were generated using BoxShade. *Pf*TrxR shows a 40% sequence identity to the longer hTrxR2 and a 42% identity to the shorter hTrxR1. It has a 77%–80% identity to the five other listed *Plasmodium* species (Table 1). The TrxR in *P. berghei* has been localized to the cytosol [30]. This could explain its slightly greater identity to hTrxR1 than hTrxR2. As described in Figure 2 the CVNVGC active site is conserved for all six species of *Plasmodium* and human isoenzymes. Conversely, the GCGGGKC C-terminal is conserved in all six *Plasmodium* species but is not present in the human isoenzymes. Sequences of TrxR for *P. falciparum* (*P. falc.*), *P. vivax* (*P. viv*), *P. yoelli yoelli* (*P. yoel.*), *P. berghei* ANKA (*P. berg*), *H. sapiens* 1 (*H. sap1*), *H. sapiens* 2 (*H. sap*2), *P. cynomolgi* strain B (*P. cyn*), and *P. knowlesi* strain H (*P. know*) were obtained from the National Center for Biotechnology Information (NCBI) protein databank.

**Table 1:** Amino acid sequence identities between *Plasmodium sp*. TrxRs the human cytosolic and mitochondrial isoforms.

	*H. sapiens* (hTrxR1)	*H. sapiens* (hTrxR2)	*P. falciparum*	*P. vivax*	*P. yoelii yoelii*	*P. berghei* ANKA	*P. knowlesi* Strain H	*P. cynomolgi* Strain B
Accession Number	AAB35418	AAD19597	CAA60574	EDL45043	EAA21839	XP_679935	XP_002258509	XP_004221759
Number of amino acids	497	524	541	546	638	542	623	628
Identity to *P. falciparum* (%)	42	40	100	77	79	79	79	80
Identity to *H. sapiens* (hTrxR2) (%)	54	100	40	41	41	40	41	41
Identity to *H. sapiens* (hTrxR1) (%)	100	54	42	41	42	42	41	41

### 3.3. Correlation of Enzymatic Inhibitory Activity between PfTrxR and the TrxRs of Other Plasmodium Species

#### 3.3.1. Selective Inhibitory Activity toward *Pf*TrxR *versus* hTrxR

Winkler, *et al.* created aculeatin analogues with Michael acceptor inhibitors and tested their effects on *Pf*TrxR activity. Michael acceptor groups have been described as capable of covalently binding to TrxR. Consistent with a mechanism of covalent modification for TrxR inactivation, all of these analogs, including (–)-aculeatin A required pre-incubation with TrxR before any inhibitory effect could be detected. Assays based on Trx reduction by a couple of aculeatin analogs consistently showed greater extents of inhibition than those recorded using DTNB but not for (–)-aculeatin. The mentioned aculeatin analogs were able to reduce the activity of the C-terminal active site with only marginal effect on the FAD active site. This mechanistic behavior suggested the involvement of other plasmodial target apart from the classical Michael acceptors. These data suggest that the C-terminal redox center is the target for TrxR modification by (–)-aculeatin [31].

Andricopulo, *et al.* have discovered that, despite of high reactivity of hTrxR at the selenocysteine active site, there are compounds that can selectively inhibit *Pf*TrxR. Thus, an inhibitor of *Pf*TrxR is a plausible antimalarial in humans because the inhibitor is less reactive towards hTrxR. The selective inhibitor of *Pf*TrxR is believed to interact with other structures or amino acid residues of the enzyme, such as the intersubunit region (interactive cavity), as opposed to the conserved C-terminal redox center [21].

#### 3.3.2. Selective Inhibitory Activity toward *Pf*TrxR *versus* Other *Plasmodium* Species TrxRs

Theobald, *et al.* have generated a toolbox of novel, recombinant reagents and assays to explore inhibition of TrxR as the potential mechanism of action of one of the subsets of compounds recently identified through phenotypic screening, the Tres Cantos antimalarial set (TCAMS), which comprises 13533 molecules that are highly potent growth inhibitors of the *P. falciparum* [32].

Seven new inhibitors of TrxR against *P. falciparum*, *P. vivax*, *P. berghei*, and *P. yoelli* isoforms were identified. These data seem to indicate that little to no selectivity between *Plasmodium* species exists. When these inhibitory compounds were tested with respect to TrxR of the four *Plasmodium* species, they were found to show noncompetitive inhibition. It was confirmed that *Pf*TrxR shows a ping-pong mechanism with its substrate and it is hypothesized that these inhibitors could prevent the dithiol-disulfide exchange in the C-terminal domain of the subunit, which would prevent the solvent exposure needed to reduce Trx [32]. All these inhibitors showed <20% cytotoxicity at 10 µM using human cell cytotoxicity data generated with the human line cell HepG2.

Assessment of the specificity of these new inhibitors remains unclear given the absence of studies of the atomic-resolution structure of *Plasmodium species* TrxR either alone or in complex with one or more of them. These studies will help to rationalize the findings in the next level of testing in animal models. This will be particularly challenging when it has been suggested that the rodent malaria parasite *P. berghei* does not depend on TrxR in its murine host [33]. The lack of essentiality of *P. berghei* TrxR in rodent models should be closely observed when testing the inhibitors due to high redundancies and multiple back-ups in the redox system that most likely ensure the survival upon loss of individual components [34].

So far, the bottleneck in finding new druggable inhibitors of *Pf*TrxR is the presence of suicide substrates, a subgroup of mechanism-based inhibitors among the most of the active compounds found in literature [35]. Rational drug design approaches are needed to overcome this obstacle of mechanism-based suicide inhibitors for instance a sort of prodrugs needed to be activated by the C-terminal arm before blocking the FAD active site and hence the entire electron transfer chain.

### 3.4. Essentiality of TrxR in Plasmodium falciparum and Other Plasmodium Species

Since *P. falciparum* lacks glutathione peroxidase and catalase, the thioredoxin systems are essential for maintaining redox homeostasis and antioxidant defense [8]. *Pf*TrxR’s substrates, *Pf*Trx1 in its cytosol and *Pf*Trx2 in its mitochondria, are necessary electron donors in these systems [21]. Oxidative stress is higher than usual in *Pf* erythrocytes due to ingestion of host cell hemoglobin and the subsequent release of free heme. This leads generation to superoxide anions that are further reduced to H_2_O_2_ by superoxide dismutase, and *Pf*TrxR systems must then reduce H_2_O_2_ to H_2_O to complete the antioxidant defense. The *P. falciparum* peroxiredoxins, particularly 2-Cys peroxiredoxin *Pf*Trx-Px1 reduces hydrogen peroxide. *Pf*Trx is responsible for reducing the peroxiredoxin back to the active form and then *Pf*TrxR must in turn reduce Trx back to the active form [36]. In a study conducted by Kranjski, *et al.*, blood stage forms of *P. falciparum* were genetically modified by inserting a TrxR2 knockout construct via transfection. Attempts to generate viable *P. falciparum* parasites in the erythrocytic stages with the TrxR2 null mutants were unsuccessful, indicating that TrxR2 knockout has a lethal affect for *P. falciparum* [37]. TrxR knockout of *P. berghei* was prepared by Buchholz, *et al*. The blast analysis showed that the *Pf*TrxR is homologous to TrxR 2, same is true with TrxR from *P*. *vivax* and *P.* *berghei*. Mice were infected with *P. berghei* in which *Pb*TrxR had been replaced with a null mutant, pTrxRRep. Results indicate that *Pb*TrxR is essential for optimal growth and viability, but viable parasites were still obtained when *Pb*TrxR was not present [33]. Interestingly, these two results are not in agreement. The differing conditions of the two experiments could contribute to the results; the *Pb*TrxR knockout parasites were tested *in vivo* using murine models while the *Pf*TrxR knockout was not tested *in vivo*. The host’s antioxidant functions under *in vivo* conditions and/or differences between antioxidant functions of mouse and human erythrocytes may also contribute to these differences.

### 3.5. Animal Models Available to Test PfTrxR Inhibitors

Murine models, or mice models, are the most commonly used first step in *in vivo* malaria drug testing, despite the apparent limitations of truly reflecting a malaria infection within a human [38]. Mice are non-primates that are normally unable to be infected by the *Plasmodium* species that affect humans. In one mouse model by Badell, *et al.* the (bg/bg xid/xid nu/nu) BXN mice were immunocompromised in order to sustain *P. falciparum*-parasitized human erythrocytes *in vivo*. The group experimented using different methods to best suppress the immune response of the mouse to sustain AB human red blood cells and parasitemia. One method was sub-lethal irradiation with cyclophosphamide or injecting di-chloromethylene biphosphonate-(CI, MBP)-encapsulated liposomes which target tissue macrophages and anti-PMN monoclonal antibodies in the mouse. A combination of the two resulted in parasitemia of 3%, but values fluctuated greatly as the immune response still reacted despite measures to suppress or the mouse died [39]. This presents that *P. falciparum* can be modeled promisingly in mice and, therefore, vaccines and antimalarials can be tested. Murine models could potentially also be used to model *P. vivax* [38,40]. Another model of *P. falciparum* parasitemia in BXN mice was developed by Moreno, *et al.*, who used di-chloromethylene diphosphonate (Cl2MDP) and NIMP-R14 monoclonal antibody to reduce tissue macrophages and blood leukocytes in the mice to sustain parasitemia for several weeks, with rare deaths occurring. Human AB or A+ blood types that were infected with *P. falciparum* African NF54 strain and the Thailand T24 strain were engrafted into the mice, whose peripheral blood then became 85%–99% human red blood cells. While parasitemia percentages were still relatively low and some fluctuation observed, maintenance of infection was sustained for up to two months. The model showed a possibility of accepting multiple isolates, and could be used to determine drug susceptibility of a human parasite, pharmacokinetics, and toxicology [41]. Validation of the usefulness of murine models has been established through the identification of antimalarials currently in clinical use. Identification of mefloquine [42], halofantrine [43], and artemisinin derivatives as antimalarials [29,43,44] was done through use of rodent models, indicating that there is correlation and ability to predict the response to treatments by infected humans. An example of the method(s) used to infect and study *in vivo* *P. berghei*, *P. yoelii*, and/or *P. chabaudi* infection *in vivo* in a murine model was established by Helmby, *et al.* [45]. *In-vivo* *P. berghei* infections in mice do not have to be immunocompromised because these species of *Plasmodium* naturally infect mice and, depending on type of experiment, ICR (outbred stock) mice, CD-1 mice, or National Medical Research Institute (NMRI) mice can be used [10,45]. A *P. berghei* mouse malaria models for evaluation of test compounds for parasitemia suppression, cure or improvement of survival of infected mice has also been described [46].

#### Primate Models

Use of a primate model after a murine model confirms the results of drug and/or vaccine efficacy in rodents and is a better prediction of any effects on humans. The owl monkey, *Aotus trivirgatus*, and *Saimiri* primate models are capable of sustaining *P. falciparum* and *P. vivax* infections. These species are well characterized and provide a more clear relationship between human infection and antimalarial response than in murine models, making the use of primate models the stage before clinical trials [40,45]. *P. vivax* can be modeled using *P. cynomolgi* infection in rhesus monkeys due to parallel symptoms. Infecting monkeys with a *Plasmodium* species can be done through the method outlined by Collins [45]. *P. cynomolgi* is a more useful model species for studying *P. vivax* than *P. knowlesi* since 217 genes are exclusive to *P. cynomolgi* and *P. vivax* while only 17 genes are exclusive to *P. knowlesi* and *P. vivax* [4]. *P. cynomolgi* is the only surrogate model available for testing the compound for antirelapse radical curative activity against hyponozoites [47]. Choice of animal model depends upon cost per test, rate of testing needed, reproducibility of efficacy of the antimalarial, the known sensitivity of the drug, and other factors relevant to the research.

### 3.6. PfTrxR and Antimalarial Drug Resistance

*P. falciparum* has acquired resistance against almost all antimalarial drugs presently under clinical use [48]. Several reports have shown significant role of glutathione (GSH) and associated functions in drug resistance of *P. falciparum*. A recent report has shown an increased transport of GSH into the parasite digestive vacuoles in *P. falciparum* strains with a mutated chloroquine resistant transport, which confers the CQ resistance [49]. The multidrug resistance-associated protein (*Pf*MRP) was reported to be involved in efflux of GSH; which might contribute to parasite responses to multiple antimalarial drugs [50]. The inhibitors of TrxR have been shown to be active against susceptible as well drug resistant malaria parasites [21]. A direct role of Trx and TrxR in antimalarial drug resistance has not been investigated. In view of a close connection between GSH and Trx pathways and the prominent role of GSH in antimalarial drug resistance; the role of Trx pathway in antimalarial drug resistance may be investigated. A recent study showed that overexpression and nuclear translocation of thioredoxin-1 (Trx-1) are closely associated with hypoxia related drug resistance in HeG2 cells through the regulation of the metabolism by the oxidative stress response to glycolysis [51]. A curcumin analog sensitized the cisplatin-resistant A549 cells to cisplatin by inhibiting TrxR [52]. Both GSH and Trx metabolism have widely implicated in resistance of cancer cells to chemotherapy [53]. TrxR has been shown to be involved in activation of 5-nitroimidazole and linked to 5-nitroimidazole resistance in *Giardia lamblia* [54]. Overexpression of TrxR has also been linked to Trx functions that modulate drug-specific cytotoxic responses [55].

## 4. Future Studies

The overview on *Pf*TrxR and other malaria species presented above clearly underline the necessity of Trx related functions for survival of the *P. falciparum* within the host erythrocytes. The malarial Trx system functions in tandem to the glutathione system. Further testing on whether or not viable parasites of *P. falciparum* and *P. berghei* could be created under similar TrxR knockout conditions is needed to provide a more definitive answer as to whether or not TrxR is essential for the survival of the human and rodent species. Further TrxR knockout testing with other *Plasmodium* strains would also be useful in understanding the essentiality of TrxR across *Plasmodium* species. The crystal structure has been determined only for *P. falciparum*; therefore, crystallization of the TrxRs of other *Plasmodium* species could provide information about structural and active site similarities between *Plasmodium* species and the human form. Also, the crystal structure of *Pf*TrxR may be used as a template to determine structures of TrxR from other malaria species by homology modeling. Striking similarities and sequence homologies between TrxR form different malaria species is important and indicate that the rodent malaria model would be suitable testing the inhibitors of *Pf*TrxR with potent antimalarial activity *in vitro* in phenotypic *P. falciparum* screening.
